# *ABCA3* Surfactant‐Related Gene Variant Associated Interstitial Lung Disease in Adults: A Case Series and Review of the Literature

**DOI:** 10.1002/rcr2.70304

**Published:** 2025-07-30

**Authors:** James Nolan, Jonathan Rodgers, John A. Mackintosh

**Affiliations:** ^1^ Department of Thoracic Medicine The Prince Charles Hospital Brisbane Australia; ^2^ School of Medicine The University of Queensland Brisbane Australia; ^3^ Genetic Health Queensland The Royal Brisbane and Women's Hospital, Genetics Health Brisbane Australia

**Keywords:** ABCA3 gene, interstitial lung disease, pulmonary fibrosis, surfactant‐related gene variant

## Abstract

Surfactant‐related gene (SRG) variants are a rare but increasingly recognised cause of interstitial lung disease (ILD) in adults. Lung disease due to pathogenic variants in the adenosine triphosphate (ATP) binding cassette subfamily A member 3 (*ABCA3*) gene has been extensively described among infants and children but is rarely described in an adult population. The rarity and heterogeneity of lung disease due to *ABCA3* variants raise significant challenges in recognition, diagnosis and management. In this case series we present three unique adult cases of ILD secondary to compound heterozygous *ABCA3* variants, review the literature to provide an overview of this disease in an adult population and highlight the role for early genetic testing in young adults presenting with unusual ILD.

## Graphical abstract

Surfactant‐related gene variants are a rare but increasingly recognised cause of interstitial lung disease in adults. Lung disease due to pathogenic variants in the ABCA3 gene has been extensively described among infants and children but is rarely described in an adult population. In this case series, we present three unique adult cases of ILD secondary to compound heterozygous ABCA3 variants, review the literature to provide an overview of this disease in an adult population, and highlight the role for early genetic testing in young adults presenting with unusual ILD.

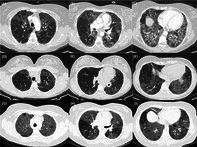

## 1 Introduction

Surfactant‐related gene (SRG) variants predominantly cause respiratory disease in the paediatric population but are increasingly recognised as a rare cause of interstitial lung disease (ILD) in adults [1, 2]. Infants with biallelic loss of function variants in the adenosine triphosphate (ATP) binding cassette subfamily A member 3 (*ABCA3*) gene present with neonatal respiratory failure and death by 1 year of age [1, 3]. Other combinations of biallelic ABCA3 variants (e.g., missense, splicing, or in‐frame insertion/deletions) resulting in adult ILD have been reported in case reports and small cohort studies, but clinical information is limited [4, 5]. Herein, we describe three unique adult cases of ILD secondary to *ABCA3* variants and review the literature to provide clinical and radiological clues to establishing a diagnosis and discuss management.

## 2 Case Series

### 2.1 Case 1

**FIGURE 1:**
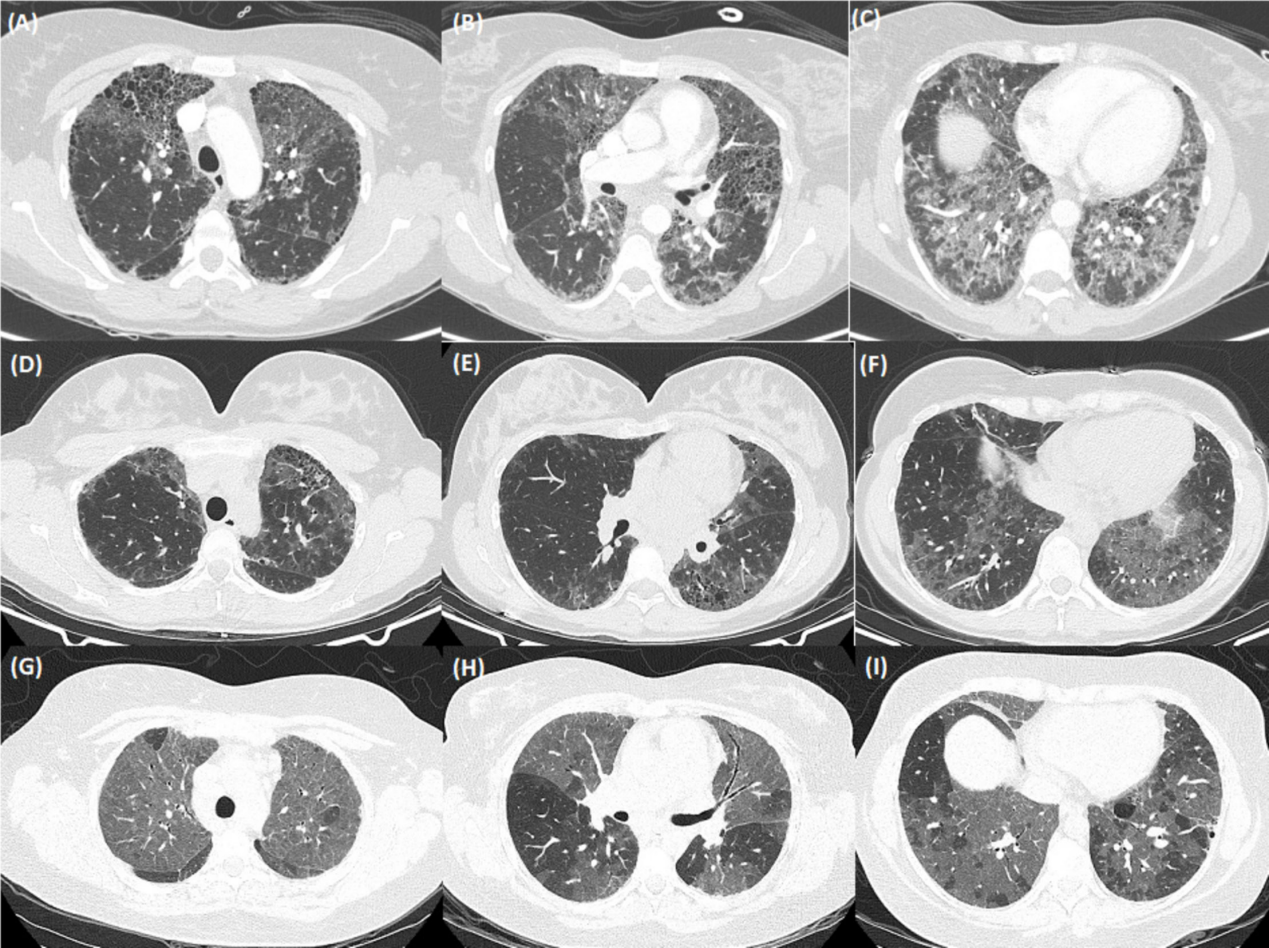
Case series of three females with ILD secondary to ABCA3 variants. Representative CT slices at presentation from (A–C) Case 1, (D–F) Case 2, and (G–I) Case 3.

**FIGURE 2:**
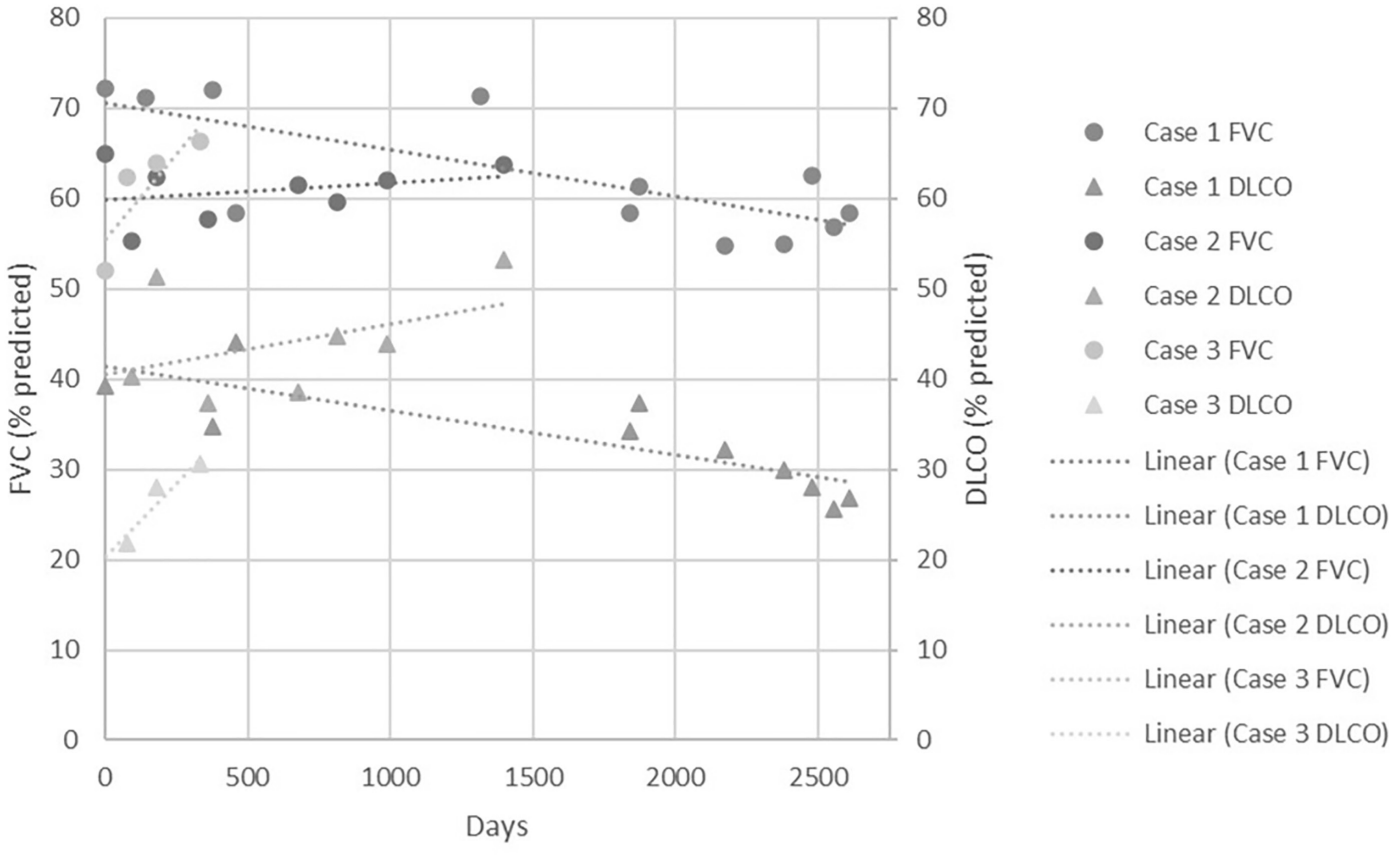
Lung function trajectory from first presentation.

A 39‐year‐old female was admitted to hospital with urosepsis, at which time she was noted to have clubbing, with incidental fibrotic lung changes on abdominal computer tomography (CT) imaging. She had a medical history of hypertension, depression, and chronic back pain, with a nominal diagnosis of asthma and an active smoking history of 25 pack‐years. Dedicated pulmonary imaging showed extensive honeycombing cystic formation in a non‐specific distribution (Figure 1). Spirometry was moderately restrictive with FVC 2.75 L (72% predicted) and severely reduced gas diffusion with DLCO 39% predicted (Figure 2). A wedge biopsy was undertaken with histology favouring fibrosing non‐specific interstitial pneumonia (NSIP) with possible early usual interstitial pneumonia. During a subsequent hospitalisation with acute hypoxic respiratory failure, interval CT demonstrated the development of extensive ground glass opacification (GGO) and treatment with azathioprine and oral steroids was initiated. Over the following 4 years, the patient suffered frequent hospitalisations with acute and infective exacerbations of ILD. Maintenance immunosuppression was changed to mycophenolate, pulmonary function continued to decline and domiciliary oxygen was supplied for progressive hypoxia. Lung transplantation assessment was prevented by steroid‐associated obesity and intermittent smoking. Six years after initial ILD diagnosis, the patient was hospitalised with acute decompensated pulmonary hypertension. Two years later, genetic testing of a panel of genes associated with ILD identified one pathogenic and one likely pathogenic variant in *ABCA3* (c.875A>T, p.(Glu292Val) and c.4747C>T, p.(Arg1583Trp); NM_001089.3 respectively) [3]. An additional variant of uncertain significance (VUS), ABCA3 c.235G>A, p.(Glu79Lys), was also identified in the gene, which is predicted to be deleterious by most in silico tools. Parental samples were not available for phasing variants. In the context of progressive pulmonary decline, lung transplantation was revisited, but during assessment, the patient was diagnosed with extensive stage small cell lung cancer. Due to deteriorating performance status, best supportive care was provided and she died 3 months later, 9 years after initial identification of ILD.

### 2.2 Case 2

A 39‐year‐old female was referred to the outpatient clinic with 10 years of progressive dyspnoea. She had a nominal diagnosis of asthma and was the mother of two children delivered without complication. There was no family history of pulmonary disease. On initial review, she was noted to be clubbed but with normal SpO_2_ on room air. Spirometry demonstrated moderate restriction with FVC 1.83 L (55% predicted) and severely reduced gas diffusion with DLCO 40% predicted (Figure 2). CT thorax demonstrated patchy GGO surrounding numerous thin‐walled cysts with some areas of co‐existent cylindrical destruction bordering on honeycombing (Figure 1). An ILD gene panel revealed a pathogenic frameshift variant (*ABCA3*:c.3997_3998del; p.(Arg1333Glyfs*24)) and a pathogenic missense variant (*ABCA3*:c.875A>T; p.(Glu292Val)). Parental testing confirmed that each parent was a carrier of one of the variants, and the patient was therefore compound heterozygous. Antifibrotic therapy, nintedanib, was initiated, but radiological progression was noted on serial imaging over 2 years. Hydroxychloroquine (HCQ) was subsequently prescribed, with 1‐year interval radiology showing reduction in GGOs, but progressive focal fibrotic changes. However, clinical progress, spirometry, and gas diffusion remained relatively stable over a further 3‐year period, and the patient continues to work full‐time in a clerical role.

### 2.3 Case 3

A 30‐year‐old female was referred to their local hospital service with 3 years of progressive dyspnoea. She was a never smoker, without significant past medical history and the mother of three children born without complication. There was no family history of pulmonary disease. Notable reported exposures were household mould and an Alexandrine parrot of 10 years. At the time of initial assessment, she was noted to be clubbed with resting SpO_2_ 91% on room air. Spirometry demonstrated moderate restriction with FVC 1.98 L (62% predicted) and severely reduced gas diffusion with DLCO 22% predicted (Figure 2). A 6‐min walk test (6MWT) showed significantly reduced walk distance at 330 m and marked exertional hypoxia with nadir SpO_2_ 74%. CT of the thorax revealed extensive GGO with mosaic attenuation, areas of minor subpleural cysts, prominent mediastinal and hilar lymphadenopathy, and dilatation of the pulmonary arteries (Figure 1). Autoimmune serology was non‐contributory. Bronchoscopy was performed with bronchoalveolar lavage cytology demonstrating 9% lymphocytes and 7.5% eosinophils; transbronchial lymph node aspirate showed no abnormality. She subsequently presented to a peripheral hospital 3 months later with progressive dyspnoea and hypoxic respiratory failure. Interval CT imaging was stable. Broad‐spectrum antibiotics were administered followed by methylprednisolone 1000 mg intravenous (IV) daily for 3 days, then oral prednisolone 50 mg daily without benefit. The patient was subsequently transferred to a tertiary centre. Echocardiogram demonstrated a mildly dilated right ventricle (RV) with preserved systolic function and mildly dilated main pulmonary arteries. Borderline elevated pulmonary pressures with a mean pulmonary artery pressure of 21 mmHg were found on right heart catheterisation. Genetic testing was requested, and treatment withHCQ 200 mg twice daily was initiated. Domiciliary oxygen was arranged. The results of the 40 gene ILD panel demonstrated a pathogenic missense variant (c.875A>T, p.(Glu292Val)) and a VUS (c.2063 T>C, p.(Leu688Pro)) in *ABCA3*. Parental samples were not available for phasing variants. While a VUS does not represent a genetically confirmed diagnosis, the available molecular evidence leant towards the variant being pathogenic and her presentation was consistent with an SRG‐related condition. Steroids were weaned to cessation; HCQ was continued and the patient was referred for lung transplantation assessment.

## 3 Discussion

Lung disease due to *ABCA3* variants is inherited in an autosomal recessive fashion. Over 300 *ABCA3* pathogenic variants have been identified. Compound heterozygosity is reported in more than three‐quarters of patients, with a familial history of ILD in approximately a quarter of cases [4, 6]. Pathogenic variants in *ABCA3*, which encodes a lipid transporter protein crucial for surfactant production in alveolar type II cells, result in disrupted surfactant homeostasis and progressive ILD [7]. Not all variants result in complete loss of function, and there is an increasing cellular‐based understanding of ABCA3 variants beyond that of intracellular protein mis‐trafficking and lipid transportation dysfunction [1, 2, 8]. Disease is predominantly associated with lethal neonatal respiratory distress and paediatric ILD, with 5‐year survival less than 20% [1, 9]. There are rare case reports of children surviving into adulthood, and across the literature, fewer than 50 reports of ABCA3‐associated ILD diagnosed in adults [1, 4, 10].

While adult diagnosed disease appears to be a heterogenous group, with highly variable disease characteristics, there are some emerging trends within the literature [5]. Onset of disease in SRG related adult ILD is difficult to ascertain due to disease rarity but appears to clinically occur at a younger age (median 45 years) than more recognised causes such as idiopathic pulmonary fibrosis (median 65 years). ABCA3 associated ILD presenting in an adult population appears to have an even earlier age of identification (median age 31.5 years), with a possible female predominance [4].

Almost all patients present with dyspnoea and over half with a non‐productive cough, 80% have crackles on initial pulmonary examination and clubbing is reported in two‐thirds. Over one‐quarter of reported cases were current or former smokers [4]. Respiratory function tests in ABCA3 pathogenic variant‐related ILD demonstrate a moderate restrictive pattern in almost all patients at the time of diagnosis, with a moderate to severe reduction in gas diffusion. These findings are generally more severe than in other SRG‐related ILD at presentation; however, progressive annual decline occurs at a lower rate [4].

Radiological findings are most commonly that of an unclassifiable ILD with GGO predominance. Pulmonary cysts are reported in over 90%, lymphadenopathy, reticulation and fibro‐elastosis features in over 80%, with honeycombing a rare feature [4, 5, 11, 12]. It has been hypothesised that imaging follows a progression from GGO to cystic disease and reticulations prior to the establishment of fibrosis [5]. Histopathology is predominantly NSIP, with the presence of mild alveolar epithelial type II cell hyperplasia and increased alveolar macrophages [4, 11].

Overall survival for patients with *ABCA3*‐related disease is unknown, but there appears to be a better prognosis and median survival than other SRG‐related ILD, which has been reported as 10 years from diagnosis. ILD exacerbations occur in just over one‐third of patients over 10 years, with lower rates of reported pulmonary hypertension than in other SRG disorders [4]. There is a recognised increased risk of lung cancer in individuals with SRG‐ILD and growing identification of the role of surfactant‐associated gene variants in individuals with lung cancer [13, 14, 15]. However, while lung cancer was identified in Case 1, noting a significant smoking history, there is no known association between ABCA3 pathogenic variant‐related ILD and lung cancer. Given the rarity of this disease and increasing identification of association with other surfactant‐associated gene variants, further consideration of a potential link is required.

Treatment for paediatric *ABCA3*‐related ILD traditionally involves immunomodulatory drugs, which have been shown to provide initial improvements in FVC in adult patients, but minimal sustained benefit [16]. HCQ has provided reported clinical improvement in case studies with some suggestion efficacy may be *ABCA3* variant specific [16, 17, 18]. Treatment with cystic fibrosis transmembrane conductance regulator (CFTR) potentiators ivacaftor and genistein has been trialled, with suggestion it may rescue *ABCA3* phospholipid transport protein functionality [19, 20].

Genetic screening considerations for all direct family members are recommended. It has been suggested that asymptomatic carriers of pathogenic variants should undergo yearly clinical screening and respiratory function tests with 5‐yearly chest radiology [5].

The pathogenic variant c.875A>T, p.(Glu292Val) was identified in all three cases presented herein. The allele frequency of this pathogenic variant is 0.00453 in gnomAD v4, seen 7316 times, with 20 homozygotes [1, 2]. This missense variant is a functional hypomorph resulting in moderately impaired ABCA3 function [1, 2, 21, 22]. Glu292Val appears to be the most commonly reported pathogenic ABCA3 variant among adults (Tables 1 and 2).

**TABLE 1:** Summary of published cases with bi‐allelic ABCA3 pathogenic variants diagnosed in adulthood.

Participant	Age at presentation	Sex	Variant (c.DNA)	Variant (p.protein)	Literature
1	39	F	c.875A>T	p.(Glu292Val)	This study
			c.4747C>T	p.(Arg1583Trp)	
2	39	F	c.875A>T	p.(Glu292Val)	
			c.3997_3998del	p.(Arg1333Glyfs*24)	
3	30	F	c.875A>T	p.(Glu292Val)	
			c.2063 T>C	p.(Leu688Pro)	
4	52	M	c.2891G>A	p.(Gly964Asp)	Campo [11]
			c.2891G>A	p.(Gly964Asp)	
5	52	M	c.2891G>A	p.(Gly964Asp)	
			c.2891G>A	p.(Gly964Asp)	
6	33	F	c.2125C>T	p.(Arg709Trp)	Kröner [1]
			c.3579C>G	p.(Ile1193Met)	
7^a^	35	F	c.[2921‐2922delinsCG;3079G>C]	p.[(Gly974Asp; Ala1027Pro)]	Legendre [17]
			c.[2921‐2922delinsCG;3079G>C]	p.[(Gly974Asp; Ala1027Pro)]	
8	41	M	c.875A>T	p.Glu292Val	Epaud [12]
			c.3081_3092delinsCG	p.Ser1028Valfs*103	
9	19	F	c.838C>T	p.(Arg280Cys)	Klay [16]
			c.875A>T	p.(Glu292Val)	
10	61	F	c.875A>T	p.(Glu292Val)	
			c.4451G>C	p.(Arg1484Pro)	
11	77	M	c.1675G>A	p.(Gly559Arg)	
			c.4745C>G	p.(Thr1582Ser)	
12	18	F	c.737C>T	p.(Pro199Leu)	Tsuchiya [23]
			c.596C>T	p.(Pro246Leu)	
13	18	F	c.737C>T	p.(Pro199Leu)	
			c.596C>T	p.(Pro246Leu)	
14	43	F	c.4873G>T	p.(Glu1625*)	Diesler [4]
			c.875A>T	p.(Glu292Val)	
15	41	F	c.58C>T	p.(Arg20Trp)	
			c.4706_4708del	p.(lle1569del)	
16	30	F	c.4237G>A	p.(Gly1413Ser)	
			c.4444C>T	p.(Arg1482Trp)	
17	19	F	c.2068G>A	p.(Glu690Lys)	
			c.875A>T	p.(Glu292Val)	
18	50	F	c.838C>T	p.(Arg280Cys)	
			c.4984‐2A>C	p.?	
19	42	F	c2414 + 1G>C	p.?	
			c.875A>T	p.(Glu292Val)	
20	38	M	c.3081_3092delinsCG	p.(Ser1028Valfs*103)	
			c.875A>T	p.(Glu292Val)	
21	19	M	c.4483‐4507del	p.(Val1495Cysfs*21)	
			c.875A>T	p.(Glu292Val)	
22	35	F	c.127C>T	p.(Arg43Cys)	
			c.3004G>A	p.(Gly1002Ser)	
23	19	F	c.347T>C	p.(Phe116Ser)	
			c.838C>T	p.(Arg280Cys)	

^a^ Also reported by Diesler et al. [4].

**TABLE 2:** All mutations identified among adult subjects with pathogenic variants in ABCA3 (NM_001089.3).

DNA position	Protein	Number of Subjects with Mutation	Variant classification	Mutation Type	Exon	ClinVar reference (if present)	Reference, if not on ClinVar
c.58C>T	Arg20Trp	1	Likely pathogenic	Missense	5	—	Diesler [4]
c.127C>T	Arg43Cys	1	Likely pathogenic	Missense	5	VCV001767530	
c.347 T>C	Phe116Ser	1	Likely pathogenic	Missense	6	—	Diesler [4]
c.596C>T	Pro199Leu	2	Likely pathogenic	Missense	7	VCV002982329	
c.737C>T	Pro246Leu	2	Likely pathogenic	Missense	8	VCV002982329	
c.838C>T	Arg280Cys	3	Pathogenic	Missense	8	VCV000318566	
c.875A>T	Glu292Val	11	Pathogenic	Missense	9	VCV000203381	
c.1675G>A	Gly559Arg	1	Variant of uncertain significance (VUS)	Missense	14	VCV003341964	
c.2063 T>C	Leu688Pro	1	Pathogenic	Missense	17	—	Diesler [4]
c.2068G>A	Glu690Lys	1	VUS	Missense	17	VCV003629580	
c.2125C>T	Arg709Trp	1	Conflicting classifications of pathogenicity	Missense	17	VCV000162674	
c.2414 + 1G>C	?	1	Likely pathogenic	Splice site	Intron	—	Diesler [4]
c.2891G>A	Gly964Asp	2	Likely pathogenic	Missense	21	—	Campo [11]
c.2921‐2922delinsCG	Gly974Asp	1	VUS	Missense	21	—	Legendre [17]
c.3004G>A	Gly1002Ser	1	Likely pathogenic	Missense	21	—	Diesler [4]
c.3079G>C	Ala1027Pro	1	VUS	Missense	22	—	Legendre [17]
c.3081_3092delinsCG	Ser1028Valfs*103	2	Likely pathogenic	Frameshift variant	22	—	Diesler [4] and Epaud [12]
c.3579C>G	Ile1193Met	1	VUS	Missense	24	VCV002137767	
c.3997_3998del	Arg1333Glyfs*24	1	Pathogenic	Frameshift variant	26	VCV001317554	
c.4237G>A	Gly1413Ser	1	Likely pathogenic	Missense	28	—	Diesler [4]
c.4444C>T	Arg1482Trp	1	Conflicting classifications (VUS in ClinVar, Pathogenic in Diesler)	Missense	29	VCV003375130	Diesler [4]
c.4451G>C	Arg1484Pro	1	VUS	Missense	29	—	Klay [16]
c.4483‐4507del	Val1495Cysfs*21	1	Pathogenic	Frameshift variant	29	VCV002631175	
c.4706_4708del	lle1569del	1	Pathogenic	In frame deletion	30	—	Diesler [4]
c.4745C>G	Thr1582Ser	1	VUS	Missense	31	VCV003341823	
c.4747C>T	Arg1583Trp	1	Likely pathogenic	Missense	31	—	Our study
c.4873G>T	Glu1625*	1	Likely pathogenic	Stop gain	31	—	Diesler [4]
c.4984‐2A>C	?	1	Likely pathogenic	Splice variant	Intron	—	Diesler [4]

In this series, we present three unique cases of young adult females with *ABCA3*‐associated ILD, presenting with functional limitation, markedly deranged pulmonary physiology, and grossly abnormal CT imaging. There were significant delays to definitive diagnosis, with reported symptoms from 3 to 10 years and purported diagnoses of asthma. Empiric treatment did not provide clinical benefit, with significant immunosuppressive side effects, in the instance of Case 1, contributing to a delay in transplantation assessment. Early genetic testing, in young adults presenting with unusual ILD, may improve diagnosis, treatment, and outcomes for these patients.

In conclusion, the three presented cases demonstrate the variability of clinical presentation, investigative findings, and disease progression in adult patients with *ABCA3*‐related ILD. A more comprehensive understanding of *ABCA3*‐related ILD in adults is required. This series demonstrates the potential value for prompt upfront genetic testing in young people with unusual ILD, as opposed to empiric therapy.

## Author Contributions

All authors contributed equally to the drafting of this manuscript and approve its content.

## Consent

The authors declare that appropriate written informed consent was obtained for the publication of this manuscript and accompanying images.

## Conflicts of Interest

The authors declare no conflicts of interest.

## Data Availability

Data sharing not applicable to this article as no datasets were generated or analysed during the current study.
